# Reappraising the Luminescence Lifetime Distributions in Silicon Nanocrystals

**DOI:** 10.1186/s11671-018-2785-x

**Published:** 2018-11-28

**Authors:** Matthias Jakob, Amira Aissiou, William Morrish, Frank Marsiglio, Muhammad Islam, Aras Kartouzian, Alkiviathes Meldrum

**Affiliations:** 1 Department of Chemistry, Technical University of Munich, Lichtenbergstrasse 4, 85748 Garching, Germany; 2 Department of Physics, University of Alberta, Edmonton, AB T6G 2E1 Canada; 3 Department of Chemistry, University of Alberta, Edmonton, AB T6G 2G2 Canada

**Keywords:** Silicon nanocrystals, Time-resolved spectroscopy, Frequency-resolved spectroscopy, Lifetimes, Stretched exponential, Lognormal

## Abstract

The luminescence dynamics in ensembles of nanocrystals are complicated by a variety of processes, including the size-dependence of the radiative and non-radiative rates in inhomogeneous broadened samples and interparticle interactions. This results in a non-exponential decay, which for the specific case of silicon nanocrystals (SiNCs) has been widely modeled with a Kohlrausch or “stretched exponential” (SE) function. We first derive the population decay function for a luminescence decay following exp[− (*t*/*τ)*^*β*^]. We then compare the distributions and mean times calculated by assuming that either the luminescence decay or the population decay follows this function and show that the results are significantly different for *β* much below 1. We then apply these two types of SE functions as well as other models to the luminescence decay data from two thermally grown SiNC samples with different mean sizes. The mean lifetimes are strongly dependent on the experimental setup and the chosen fitting model, none of which appears to adequately describe the ensemble decay dynamics. Frequency-resolved spectroscopy (FRS) techniques are then applied to SiNCs in order to extract the lifetime distribution directly. The rate distribution has a half width of ~ 0.5 decades and mainly resembles a somewhat high-frequency-skewed lognormal function. The combination of TRS and FRS methods appear best suited to uncovering the luminescence dynamics of NC materials having a broad emission spectrum.

**Electronic supplementary material:**

The online version of this article (10.1186/s11671-018-2785-x) contains supplementary material, which is available to authorized users.

## Introduction

Colloidal nanoparticles can be used in a manifold of applications including catalysis, medical treatments, and optoelectronic applications [1–4]. Semiconductor nanoparticles are of particular interest for light emission, photovoltaic, and photocatalytic applications [5, 6]. Silicon nanocrystals (SiNCs) are a focus of current attention owing to the tunable emission properties [7] as well as the abundance and biocompatibility of silicon [8]. In order to develop nanoparticle-based technologies, a deep knowledge of the relevant optoelectronic properties is needed, and time-resolved spectroscopy is often a valuable tool for this purpose.

The luminescence lifetimes of SiNCs are usually modeled with a stretched exponential (SE) function having the basic form exp[ − (*λt*)^*β*^], where the dispersion parameter *β* takes values between 0 and 1, *λ* is a rate parameter, and *t* is time. This function is often described as “slower than exponential” and implies an asymmetric distribution of decay rates tailing toward longer lifetimes. Once the *β* and *λ* parameters have been found by fitting a luminescence decay curve, the corresponding decay rate distribution can be approximately reconstructed [9].

The origin of the SE luminescence decay in silicon and other semiconductor quantum dots has been heavily debated in the last two decades, and the debate has continued recently [10]. Various explanations have been proposed for the appearance of the SE in the decay dynamics, including carrier tunneling and trapping in closely spaced ensembles of nanocrystals [11], the inhomogeneously broadened size distribution [12], size-dependent electron-phonon coupling [10], and a distribution of barrier heights for non-radiative recombination [13], the latter being similar to a previous suggestion for porous silicon [14]. Clearly, knowledge of the rate distribution is required for an understanding of the luminescence mechanism in SiNCs as well as in semiconductor nanocrystals more generally.

In much of the previous literature on SiNCs, the stretched exponential decay was assumed a priori, usually without analysis of other possible distributions. The SE tends to fit well visually (i.e., the best-fit line appears to match the data well “by eye”). Furthermore, in the vast majority of the previous works, e.g., [15], there is a lack of clarity about whether the population decay or the luminescence decay is actually being modeled. These are related by a derivative and one should use the correct expression in order to understand the decay timescales in the sample [16]. Also, the responsivity function of the detector can have a significant effect on the measured luminescence decay curve in SiNCs, due to the broad ensemble emission spectrum. Despite this, the responsivity has rarely if ever been taken into account, making it difficult to compare results from different investigations. Finally, no previous studies have attempted to use frequency-resolved spectroscopy (FRS) in the analysis of silicon nanocrystals. In principle, FRS permits the lifetime distribution to be extracted without assuming a model a priori.

The purpose of this paper is to establish an approach to measure, model, and interpret the luminescence dynamics of silicon nanocrystals. It is hoped that this could help to better understand the vast diversity of often contradictory results in the literature, lead to better agreement, or at least more consistency, between different measurements, and to better understand the luminescence mechanisms.

## Basic Theory

We compare three models: the stretched exponential, which is widely used for Si nanocrystals, the lognormal decay distribution, which was first applied to SiNCs recently [17], and the bimolecular decay. For any model, the emission probability density function, represented by the integral of the intensity function *g*(*t*), at time *t′* is related to the fraction of excitations remaining at *t′* according to [16].

1 $$ {\int}_0^tg\left({t}^{\hbox{'}}\right) dt=1-\frac{c_t}{c_0}, $$

where *c*_*t*_ and *c*_0_ are the number of excited NCs at time *t* and initially. The probability density function describes the fraction of photons emitted between time 0 and *t* relative to the total number of photons emitted. If the population decay follows a first-order rate equation (i.e., “monomolecular” recombination), we have *dc*_*t*_/*dt =* − *λc*_*t*_, where *λ* = 1/*τ*_*0*_, leading to the usual *c*_*t*_/*c*_0_ = exp[− *λt*] and *g*(*t*) = *λ⋅*exp[− *λt*] after taking the time derivative of both sides of Eq. 1. The derivative is necessary because the luminescence intensity measured in the window *dt′* is proportional to the change in the excited fraction over that interval.

If we consider both radiative and non-radiative rates, then we replace the total decay rate *λ* with *λ*_*R*_ + *λ*_*NR*_ so that *g*(*t*) = (*λ*_*R*_ + *λ*_*NR*_)exp[− (*λ*_*R* +_ *λ*_*NR*_*t*] = *λ*_*R*_exp[− (*λ*_*Ri* +_ *λ*_*NR*_)*t*] + *λ*_*NR*_exp[− (*λ*_*R* +_ *λ*_*NR*_)*t*] in which only the first term is measurable, yielding a measured intensity for time-resolved spectroscopy (TRS) given by

2 $$ g(t)={\lambda}_R\exp \left[-\left({\lambda}_R+{\lambda}_{NR}\right)t\right]. $$

The decay function used to fit the data, *I*_*t*_ = *A·*exp(− *λt*) + dc, scales with an additional arbitrary prefactor, *A*, which depends on the detection efficiency and the number of nanoparticles excited and will lead to the appropriate scale. A dc offset is usually added to the decay function as another fitting parameter.

In the case of the stretched exponential decay, the fraction of excited emitters decays according to

3 $$ \frac{c_t}{c_0}=\exp \left[-{\left({\lambda}_{SE}t\right)}^{\beta}\right]. $$

where *λ*_*SE*_ is the stretched exponential decay rate (equal to 1*/τ*_*SE*_)*.* Inserting this into Eq. 1 and taking the derivative of both sides as before yields an emission probability function given by

4 $$ g(t)={\beta \lambda}_{SE}^{\beta }{t}^{\beta -1}\exp \left[-{\left({\lambda}_{SE}t\right)}^{\beta}\right]. $$

A way to estimate the distribution of frequencies *H*(*λ*) that leads to Eq. 3 was shown using an inverse Laplace transform [9], yielding a distribution that widens with decreasing *β* and is skewed toward high frequencies.

Unfortunately, in Eq. 4, it is not possible to separate the prefactor into radiative and non-radiative parts. This means that Eq. 4 is correctly normalized only for *λ*_*NR*_ *=* 0 [16], and the lifetime distribution obtained from a PL decay curve is only understood in this way. Moreover, there is a time-dependent term in the prefactor; therefore, the population decay has a different time dependence as compared to the luminescence decay [16, 18]. In order to obtain values of *τ*_*SE*_ and *β* for the population decay from which the appropriate mean lifetimes can be extracted, one has to use Eq. 4 to model the observed decay, where we replace *g*(*t*) by the measured decay function *I*_*t*_:

5 $$ {I}_t=A{\beta \lambda}_{SE}^{\beta }{t}^{\beta -1}\exp \left[-{\left({\lambda}_{SE}t\right)}^{\beta}\right]+\mathrm{dc}. $$

In Eq. 5, a scaling parameter (which can also absorb the *β* and *λ* terms in the prefactor) and a dc offset were inserted as fitting parameters. The mean lifetime is given by

6 $$ \left\langle {\tau}_{SE}\right\rangle =\frac{\tau_{SE}}{\beta}\Gamma \left[\frac{1}{\beta}\right], $$

where *Γ* represents the Gamma function, and the mean decay time is

7 $$ \left\langle t\right\rangle ={\tau}_{SE}\frac{\Gamma \left(2/\beta \right)}{\Gamma \left(1/\beta \right)}. $$

In much previous work, it has been common to use the “standard” stretched exponential exp[− (*λ*_*SE*_*t*)^*β*^] to model the luminescence decay instead of the population decay. Accordingly, we have a normalized intensity function given by

8 $$ g(t)=\frac{\lambda_{SE}\beta }{\Gamma \left(1/\beta \right)}\exp \left[-{\left({\lambda}_{SE}t\right)}^{\beta}\right]. $$

Equation 8 is normalized so that integration between *t* = 0 and ∞ is equal to 1. The corresponding fitting model is simply

9 $$ {I}_t=A\exp \left[-{\left({\lambda}_{SE}t\right)}^{\beta}\right]+\mathrm{dc}. $$

Equation 9 is widely applied and often fits the SiNC luminescence data quite well, despite the fact that (like Eq. 4) Eq. 8 is strictly normalized for an absolute quantum efficiency (AQY) of 100%. An often-overlooked point is the fact that one cannot extract *τ*_*SE*_ (= 1/*λ*_*SE*_) and *β* from the luminescence decay modeled by Eq. 9 and use them to calculate the mean times with Eqs. 6 and 7. Essentially, Eqs. 4 and 8 are different intensity decay models and one should expect different population decay functions, mean times, and decay rate distributions.

In order to find the population decay that would lead to an intensity function given by Eq. 9, we apply the same process we did to get from Eq. 4 to Eq. 5, but in reverse, that is:

10 $$ \frac{c_t}{c_0}=1-\frac{\lambda_{SE}\beta }{\Gamma \left(1/\beta \right)}{\int}_0^t\exp \left[-{\left({\lambda}_{SE}t\right)}^{\beta}\right]\cdot \mathrm{dt}. $$

After several steps, the solution to Eq. 10 is

11 $$ \frac{c_t}{c_0}=\frac{1}{\Gamma \left(1/\beta \right)}\Gamma \left[1/\beta, {\left({\lambda}_{SE}t\right)}^{\beta}\right]. $$

Equation 11 is the population decay obtained from the intensity decay given by Eq. 8. Finding the mean lifetime in the usual way leads to

12 $$ \left\langle {\tau}_{SE}\right\rangle ={\tau}_{SE}\frac{\Gamma \left(2/\beta \right)}{\Gamma \left(1/\beta \right)} $$

and a mean decay time of

13 $$ \left\langle t\right\rangle ={\tau}_{SE}\frac{\Gamma \left(3/B\right)}{2\Gamma \left(2/\beta \right)}. $$

Finally, the frequency distribution is (*1*/*λ*)*·H*(*λ*), where, as before, *H*(*λ*) is the distribution calculated in ref. [9] for a population decay given by Eq. 3. These results are summarized in Table 1.

**Table 1:** Formulas for stretched exponential population and luminescence decays. The approximate solution *H*(*λ*) is shown in ref. [9]

Population decay *c*_*t*_*/c*_*0*_	Intensity decay *g(t)*	Mean time constant	Mean decay time	Rate distribution
exp[−(*λ*_*SE*_*t*)^*β*^]	${\beta \lambda}_{SE}^{\beta }{t}^{\beta -1}\exp \left[-{\left({\lambda}_{SE}t\right)}^{\beta}\right]$	$\frac{\tau_{SE}}{\beta}\Gamma \left[\frac{1}{\beta}\right]$	${\tau}_{SE}\frac{\Gamma \left(2/\beta \right)}{\Gamma \left(1/\beta \right)}$	*H*(*λ*)
$\frac{1}{\Gamma \left(1/\beta \right)}\Gamma \left[1/\beta, {\left({\lambda}_{SE}t\right)}^{\beta}\right]$	$\frac{\lambda_{SE}\beta }{\Gamma \left(1/\beta \right)}\exp \left[-{\left({\lambda}_{SE}t\right)}^{\beta}\right]$	${\tau}_{SE}\frac{\Gamma \left(2/\beta \right)}{\Gamma \left(1/\beta \right)}$	${\tau}_{SE}\frac{\Gamma \left(3/\beta \right)}{2\Gamma \left(2/\beta \right)}$	*H*(*λ*)/*λ*

**Fig. 1:**
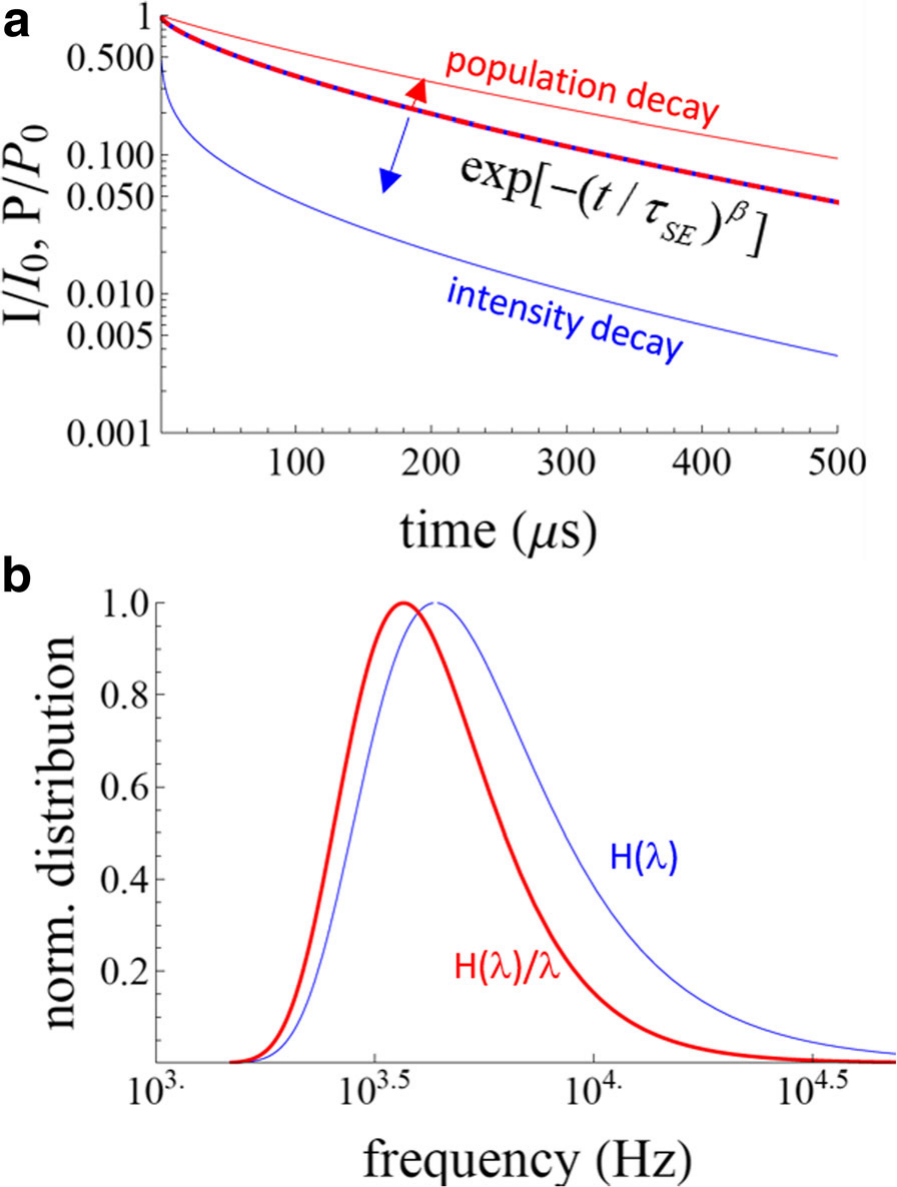
Stretched exponentials. **a** Population and intensity decays for the stretched exponential function with *τ*_*SE*_ = 100 μs and *β* = 0.7. The blue-red dashed line is exp[*−*(*λt*)^*β*^]. If this represents the population decay, then the intensity decay will be given by the blue line. If exp[*−*(*λt*)^*β*^] is the intensity decay, then the population decay is shown with the red line. **b** The corresponding rate distributions

The differences between the two SE formulas are significant (Fig. 1). In the literature, one frequently finds that the intensity decay is modeled by *A·*exp[− (*t*/*τ*_*SE*_)^*β*^] + dc (i.e., Eq. 9) and then the mean times are calculated using Eqs. 6 and 7. This appears to be mathematically incorrect, since Eqs. 6 and 7 are derived from an intensity decay given by Eq. 4, not Eq. 8. For example, taking *τ*_*SE*_ = 100 μs and *β* = 0.7, as shown in Fig. 1, for an intensity decay given by exp[− (*t*/*τ*_*SE*_)^*β*^], we find a mean time constant of 199 μs (Eq. 12), as compared to 127 μs by using Eq. 6. Similar differences are found for the mean decay times (Eqs. 7 and 13). Additionally, there is an approach known as the Higashi-Kastner method for estimating a characteristic lifetime [19], which has been applied to SiNCs as an alternative to applying the SE decay model [20, 21]. In this model, the characteristic delay time, *t*_*d*_*,* is simply taken as the peak of the decay data plotted as *I*_*t*_*·t* vs. *t*. This was suggested to be equivalent to (*1*/*β*)^*1*/*β*^*·τ*_*SE*_ obtained from Eq. 9 [20].

Alternatively, the distribution of decay rates may follow a specific *Η*(*λ*), leading to a luminescence decay given by:

14 $$ g(t)={\int}_0^{\infty}\mathrm{H}\left(\lambda \right)\cdot \exp \left(-\lambda t\right)\mathrm{d}\lambda, $$

where *Η*(*λ*) represents the frequency-dependent distribution of decay rates. Equation 14 reduces to Eq. 2 if *Η*(*λ*) is equal to the Dirac delta function *δ*(*λ − λ*_0_), or it can represent a continuous series of exponentials weighted by the selected distribution. A lognormal function seems a reasonable choice in nanocrystal systems since many nanocrystal ensembles naturally follow lognormal size distributions [22]. In order to avoid further confusion, we use the standard normalized definition of lognormal function given by:

15 $$ H\left(\lambda \right)=\frac{1}{\lambda}\cdot \frac{1}{\sigma \sqrt{2\pi }}\exp \left[-\frac{{\left(\ln \lambda -\mu \right)}^2}{2{\sigma}^2}\right]. $$

so that the measured decay function is

16 $$ {I}_t=A\cdot {\int}_0^{\infty}\left(\frac{1}{\lambda}\cdot \frac{1}{\sigma \sqrt{2\pi }}\exp \left[-\frac{{\left(\ln \lambda -\mu \right)}^2}{2{\sigma}^2}\right]\cdot \exp \left(-\lambda t\right) d\lambda \right)+ dc. $$

As with the SE function, there are only two independent variables (as well as an offset and a scaling factor). The moments are defined as usual; i.e., the median rate is given by *exp*(*μ*), the mean by *exp*(*μ* + *σ*^*2*^/2), and the most probable lifetime (the peak of the distribution) is *exp*(*μ* − *σ*^*2*^). Previously, a non-standard distribution was employed [16] (i.e., a distribution that, while valid on its own, is not the commonly accepted lognormal distribution function). Equation 14 also applies to a radiative decay distribution (i.e., AQY = 100%). In fact, it has been suggested that decay rate distributions are weighted by an (unknown) quantum efficiency function [16]. In real situations, one simply has to accept this caveat given that it is difficult or impossible to know the population distribution of non-radiative rates in the sample.

Luminescence decays may also correspond to a second-order reaction (i.e., the “bimolecular” decay) [23]. Here, the rate at which the population decays is given by *dc*/*dt* = − *λ*[*c*_*t*_]^2^, which yields a remaining fraction *c*_*t*_/*c*_0_ = (*c*_0_*λt* + 1)^−1^. Inserting this expression into Eq. 1 results in a power law decay:

17 $$ {I}_t/{I}_0=A\frac{\lambda {c}_0}{{\left(\lambda {c}_0+1\right)}^2}. $$

The bimolecular model has only one rate constant *λ* (unlike the stretched exponential and lognormal, which have distributions of rates), and there is no mean lifetime. More specifically, the time integral diverges and the mean lifetime of the second-order decay is infinite.

The “standard” SE function (Eq. 9) has been by far the dominant model used for SiNC luminescence decays, with many papers devoted to interpreting the meaning of the decay for the luminescence mechanisms. The lognormal lifetime distribution was first applied to SiNCs quite recently [17, 24, 25]. Obviously, there is little a priori reason to assume any model, and it would instead be preferable to establish the distribution of decay rates directly. This can, in principle at least, be achieved by quadrature frequency-resolved spectroscopy (QFRS), which has been applied on several occasions to amorphous silicon but not to SiNCs.

### Quadrature Frequency-Resolved Spectroscopy

The QFRS method is rather sparsely reported in the literature, mainly limited to a few studies of rare-earth-doped glasses [26–28] and amorphous silicon [29–31]. The basis of the technique is to excite the sample with a sine-wave-modulated pump beam of angular frequency *ω* and to measure the phase and amplitude of the luminescence as it attempts to track the excitation. With this setup, the quadrature component (*Q*) of a phase-sensitive detector (i.e., a lock-in amplifier) provides a direct measure of the lifetime distribution [30]. Since the amplitude of the AOM-modulated laser oscillation can be frequency-dependent, the quadrature component of the PL, *Q*_*PL*_ = *Z*_*PL*_*sin*(*Δθ*_*PL*_) has to be normalized to the amplitude of the laser oscillation, *Z*_*LA*_.

The quadrature FRS signal is complicated by the fact that a single exponential decay does not result in a delta function in the QFRS spectrum. The observed signal is in fact the convolution of the lifetime distribution with a single exponential response function given on a log scale by [31].

18 $$ {S}_{\log_{10}\mathrm{r}}=\frac{{\omega \tau}_0}{1+{\omega}^2{\tau}_0^2}, $$

Where the time constant *τ*_*0*_ = *ω*_0_^−1^. Thus, unless the decay rate distribution is several decades wide, a deconvolution has to be performed in order to extract a meaningful distribution.

## Results and Discussion

### Basic Characterization

**Fig. 2:**
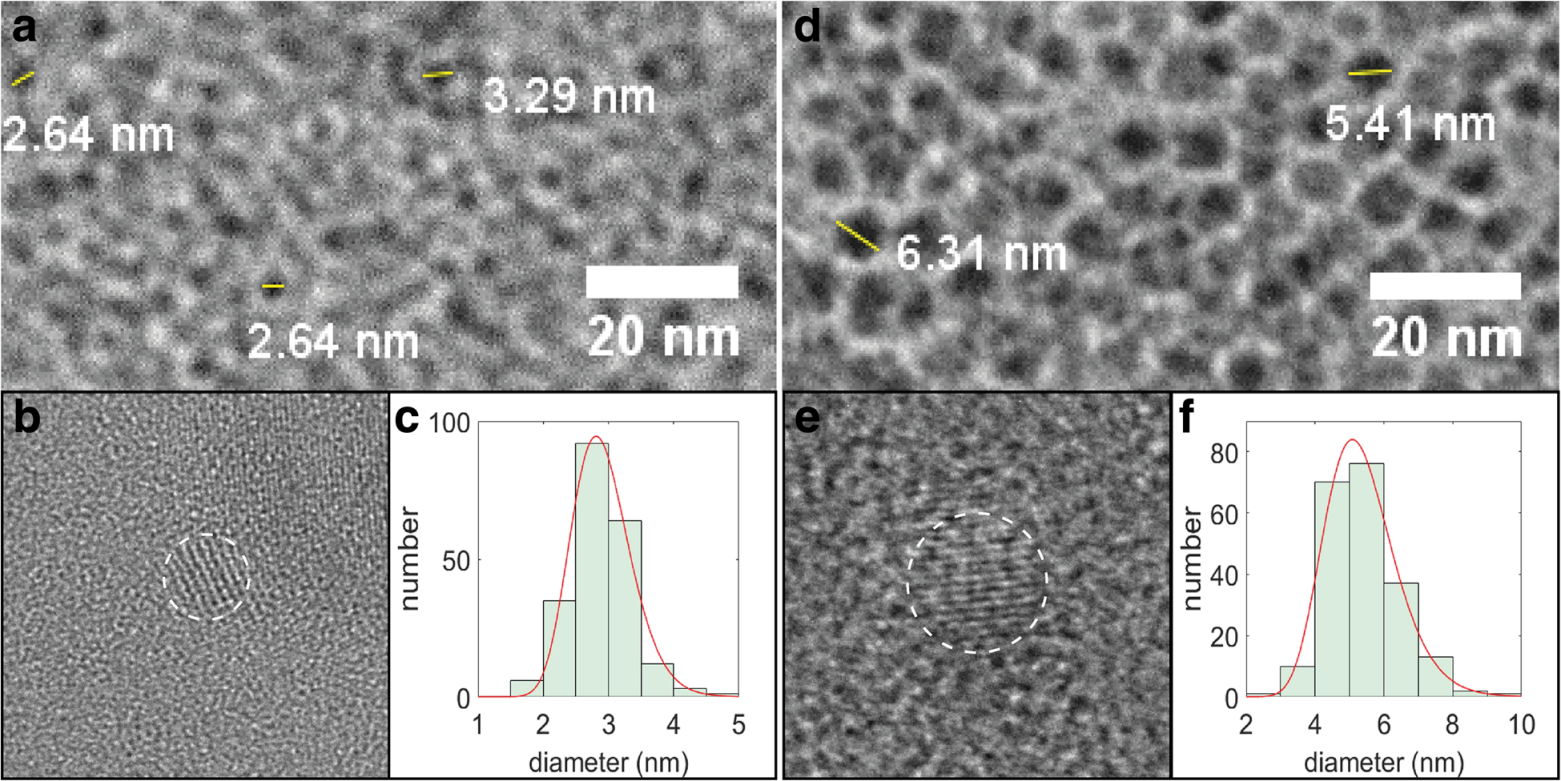
TEM images of SiNCs. **a** Bright-field, **b** high resolution, and **c** size distribution histogram for the small SiNCs. Panels **d**–**f** represent a similar set of images from the large SiNCs

Due to the low contrast associated with the SiNCs and the overlapping mottled contrast from the amorphous carbon support, computer-based particle counting algorithms using bright-field images cannot be applied and the diameters had to be estimated “by eye” using pixel counting software (sample bright-field TEM images are shown in Fig. 2a, d and the manual particle counting results were fit with a lognormal distribution (Fig. 2c, f) in order to obtain a linear mean diameter of 2.9 nm (mean and standard deviation of the natural logarithms *μ* = 1.057 and σ = 0.1555) and 5.4 nm (*μ* = 1.663 and σ = 0.1917), for 1100 and 1200 °C annealing temperatures, respectively. These samples will henceforth be referred as “small” and “large” SiNCs. The sizes were further checked by high-resolution imaging of selected NCs (Fig. 2b, e), where the lattice fringes could be used as another way to identify the NCs and estimate their diameters. The Fourier transform infrared (FTIR) spectroscopy and XPS data showed that the prepared SiNCs were successfully functionalized with dodecene; however, the small SiNCs are more oxidized than the large ones and thus show a smaller degree of functionalization (Additional file 1: Figures S1 and S2).

### Photoluminescence and Time-Resolved Spectroscopy

**Fig. 3:**
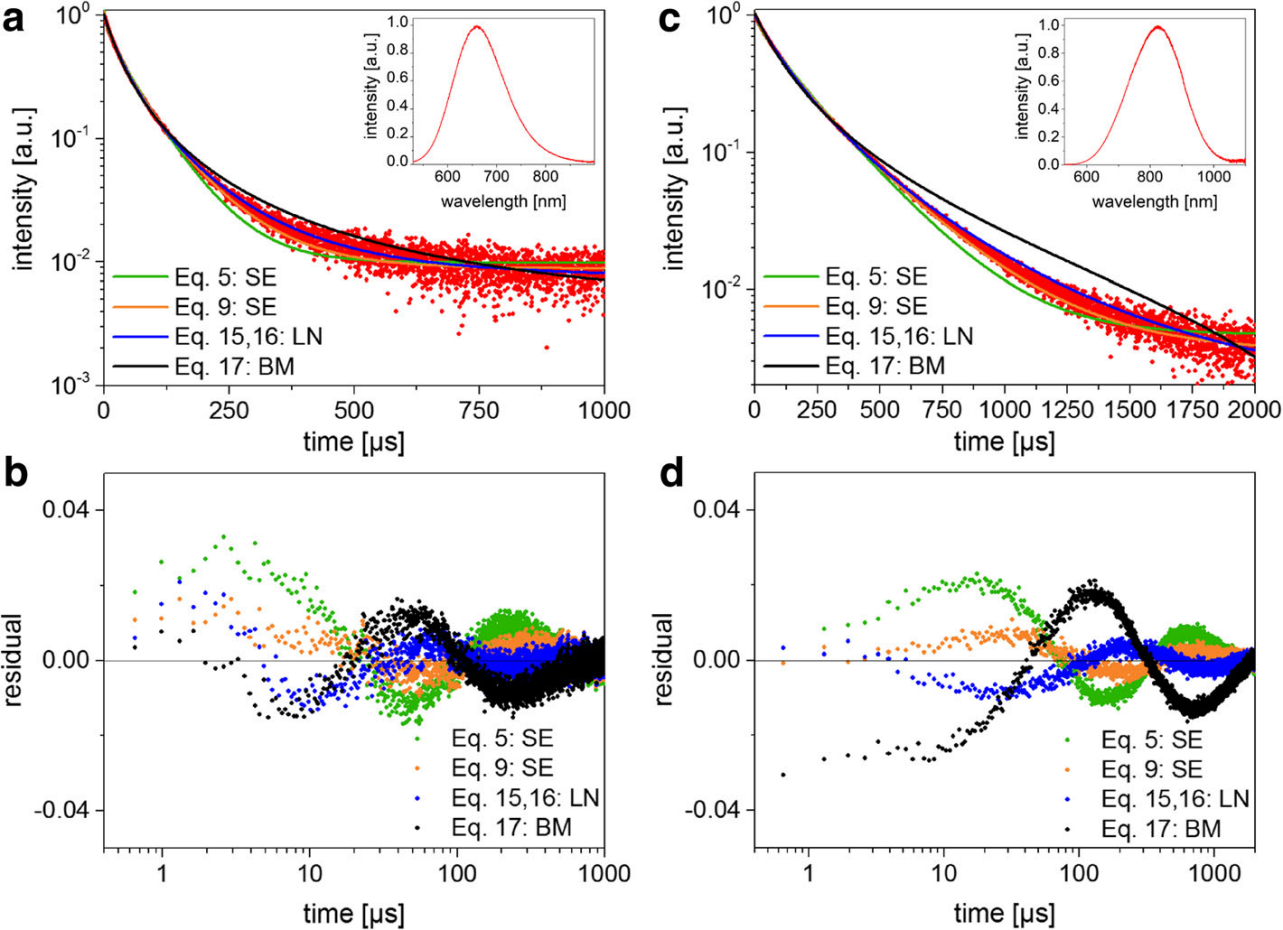
TRS data and fitting results. **a** Luminescence decays and the corresponding fitting function (*BM* bimolecular, *SE* stretched exponential, *LN* lognormal) for small SiNCs. The PL spectrum is shown in the inset. **b** Residuals plots for the fits in (**a**, **c**, **d**) show the curves and residuals for the large SiNCs.

The photoluminescence (PL) spectra were centered at 660 and 825 nm with a full-width-at-half maximum of 123 and 198 nm for small and large SiNCs, respectively (Fig. 3 insets). The indirect bandgap energies are predicted to be 1.87 and 1.37 eV according to ${E}_g\kern0.5em =\kern0.5em \sqrt{E_{g,\mathrm{bulk}}^2\kern0.5em +\kern0.5em D/{R}^2}$ [32] with *D* = 4.8 eV^2^/nm^2^ and *R* being the NC radius, which is in close agreement for the small particles but predicts a slightly smaller bandgap than obtained by the PL peak for the large ones. The AQY was 12% for the small SiNC sample and 56% for the large NCs. Independent measurements on a different system yielded 18% and 48% for the two samples, which is typical of the uncertainties in AQY measurements [33] for the different excitation and cutoff wavelengths. We hypothesize that the less curved, lower-energy surfaces of the larger NCs leads to a better surface functionalization and a smaller contribution from non-radiative surface states to the overall PL spectrum.

**Fig. 4:**
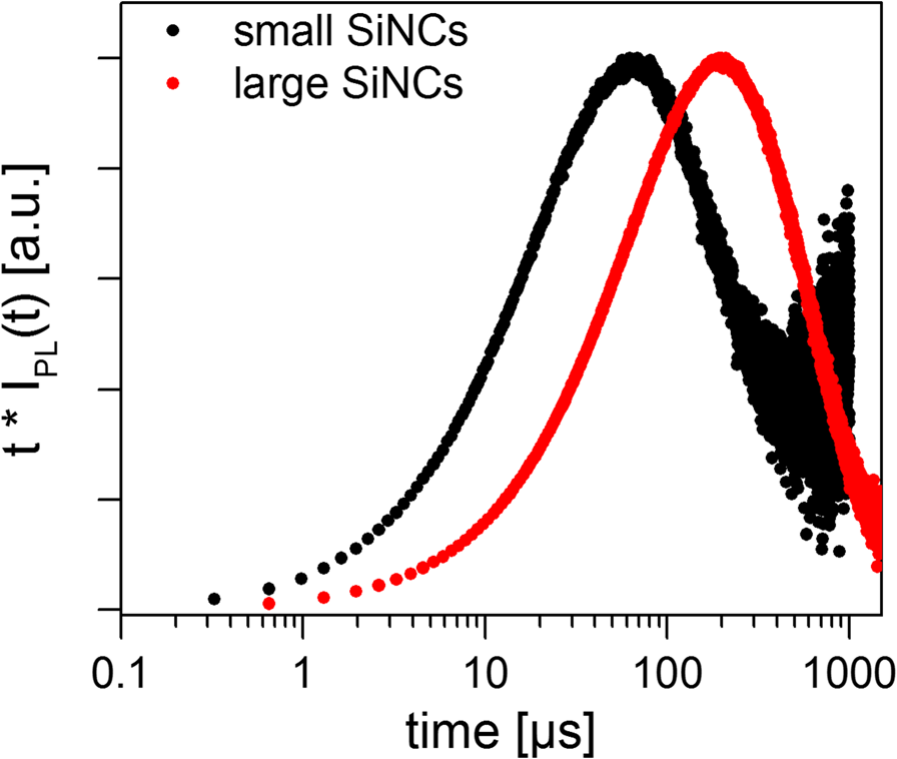
Normalized PL decay curves multiplied by the decay time (Higashi-Kastner plots) for the small and large SiNC ensembles. The peak positions represent the most dominant decay time, represented by *t*_*d*_ in Table 2

Both samples yielded a non-exponential decay, as expected on the basis of the extensive previous literature on SiNCs. The measured PL decays were fit with Eqs. 5, 9, 16, and 17 in order to test the different models using standard sum-of-squares minimization (Fig. 3). The fact that the detector responsivity is not constant over the wide NC luminescence spectrum will be discussed later. For all cases, the residuals oscillate, indicating that none of the models appears fully adequate, but the “simple” SE model (Eq. 9) and the lognormal (Eq. 16) tend toward the lowest sum of squares of the residuals. The calculated fitting parameters and mean lifetimes for the two SiNC samples are shown in Table 2, in which the means are clearly dependent on the selection of the decay model. The Higashi-Kastner method was also applied (Fig. 4) and the peak positions determined by fitting the delay time curves with a skewed Gaussian. The Higashi-Kastner method yields a time constant *t*_*d*_ quite similar to (*1*/*β*)^*1*/*β*^*∙τ*_*SE*_, with these values take from Eq. 9 as shown before [20]. The bimolecular model fits fairly poorly, consistent with isolated nanocrystals that are not heavily over-excited. It will therefore not be further discussed.

**Table 2:** Fitting parameters, mean lifetimes, and mean decay times obtained for Eqs. 5, 9, and 16 for the small and large SiNC samples. For the lognormal function (Eq. 16), the last column shows the mode (i.e., the most common lifetime). All lifetimes are in microseconds

Sample	Equation 5				Equation 9				Equation 16				HK method
	*τ*_*SE*_	*β*	〈*t*〉	〈*τ*_*SE*_〉	*τ*_*SE*_	*β*	〈*t*〉	〈*τ*_*SE*_〉	*μ*	*σ*	〈*τ*_*LN*_〉	〈*τ*_mode, *LN*_〉	*t*_*d*_
Small SiNCs	60.9	0.92	69.4	63.4	38.9	0.72	89.7	73.3	10.1	0.73	31.4	70.4	62.8
Large SiNCs	195	0.94	212	200	146	0.79	253	220	8.8	0.61	124	217	194.8

In order to estimate the number of excitons per NC on average for these measurement conditions, the excitation rate has to be calculated from the absorption cross sections, which can evidently be as high as 10^−14^ cm^2^ for these experiments [34]. Given an excitation irradiance of 4500 W/m^2^ at 352 nm and the measured peak emission rates (see following sections), the number of excitations per NC for the large and small SiNCs was estimated to be less than ~ 1 and 0.2, respectively. This suggests that the large SiNCs may be slightly over-excited. This can cause additional non-radiative effects due to the presence multi-excitons in some NCs. In order to further evaluate this possibility, the lifetime was measured as a function of excitation power; down to 2% of the values reported above. The results showed no trend and were always the same within ~ 2% (Additional file 1 Figure S3), which is close to the fitting and repeatability errors despite the low signal-to-noise ratio in the low-power measurements. Thus, the possible over-excitation of the NCs appears to have little effect on the results.

**Fig. 5:**
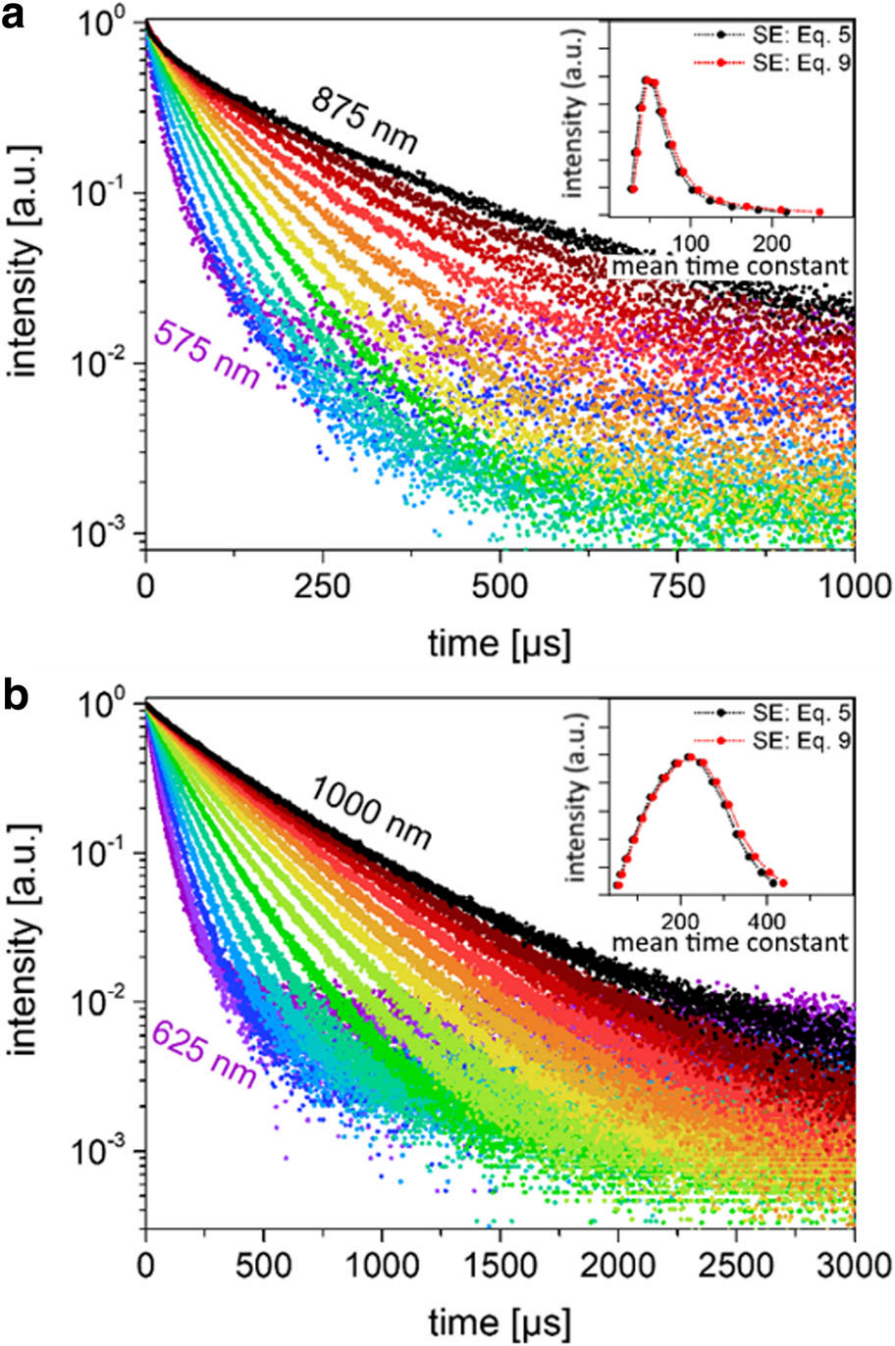
Narrow-wavelength PL decays. **a** Luminescence decays for the small SiNCs at specific emission wavelengths (3 nm FWHM) ranging from 575 to 875 nm, in 25-nm intervals. The data were fit with Eqs. 5 and 9, which yielded a nearly single exponential fit. **b** Luminescence decays at specific emission wavelengths ranging from 625 to 1000 nm for the large SiNCs measured and fitted under the same conditions. The resulting time constants for the small and the large SiNCs are given in Table 1

In order to estimate the lifetime distribution from TRS, the decays were measured over a set of fixed wavelengths using a monochromator with a ~ 3 nm bandpass (Fig. 5). Due to the low intensity, a photon-counting PMT system was used for this purpose. With effectively monochromatic radiation, there should be no difference in the decay constants measured with different detectors since there is negligible distribution of the response function over such a narrow range of wavelengths. The same trend was found for the dodecyl-terminated particles as observed for in other silicon NCs [25, 35, 36]; that is, the dispersion parameter increases closer to unity and the lifetime increases rapidly as a function of the wavelength (Fig. 5, Table 3).

**Table 3:** Wavelength dependence of the SiNCs lifetimes and fitting parameters obtained from Eq. 5 and 9. The lifetimes changed by almost an order of magnitude across the emission spectrum. The β values were above 0.8 in most cases

Small SiNCs						Large SiNCs					
λ (nm)	I_PL_	*β*_*Eq*. 5_	〈*τ*_*SE*_〉_*Eq*. 5_	*β*_*Eq*. 9_	〈*τ*_*SE*_〉_*Eq*. 9_	λ (nm)	I_PL_	*β*_*Eq*. 5_	〈*τ*_*SE*_〉_*Eq*. 5_	*β*_*Eq*. 9_	〈*τ*_*SE*_〉_*Eq*. 9_
575	0.19	0.85	27.3	0.65	30.2	–	–	–	–	–	–
600	0.45	0.89	31.7	0.71	34.6	–	–	–	–	–	–
625	0.77	0.92	37.6	0.76	40.4	625	0.08	0.92	53.1	0.76	57.7
650	0.97	0.93	44.5	0.80	47.2	650	0.15	0.94	61.2	0.82	64.7
675	0.95	0.94	53.2	0.82	56.2	675	0.26	0.96	73.9	0.86	77.4
700	0.74	0.95	62.4	0.84	65.6	700	0.39	0.96	89.8	0.87	93.5
725	0.50	0.95	73.5	0.83	77.7	725	0.55	0.97	108.5	0.88	112.8
750	0.31	0.95	86.0	0.83	91.2	750	0.70	0.97	130.9	0.89	136.0
775	0.18	0.94	102.0	0.81	109.5	775	0.84	0.97	158.3	0.89	164.6
800	0.10	0.93	123.6	0.78	135.2	800	0.94	0.98	187.2	0.90	194.2
825	0.06	0.92	150.8	0.74	168.5	825	0.98	0.98	217.1	0.91	224.3
850	0.04	0.92	182.9	0.71	210.8	850	0.94	0.98	245.7	0.92	253.0
875	0.02	0.91	217.0	0.68	258.0	875	0.81	0.98	274.1	0.91	282.6
–	–	–	–	–	–	900	0.65	0.98	301.9	0.92	311.6
–	–	–	–	–	–	925	0.44	0.98	329.6	0.91	341.1
–	–	–	–	–	–	950	0.28	0.98	358.3	0.91	372.6
–	–	–	–	–	–	975	0.16	0.98	387.5	0.89	405.9
–	–	–	–	–	–	1000	0.09	0.97	414.6	0.88	437.9

The smaller particles always had a shorter lifetime than the larger ones at the same measurement wavelength. This observation is consistent with the lower AQY of the smaller particles, indicating that the lifetime of the large NCs is less strongly governed by non-radiative processes. The large NCs are also less oxidized in comparison to the small NC sample (Additional file 1 Figure S1). Thus, while the observation of the lower AQY on the small sample is consistent with the measured shorter lifetimes, one cannot make a relative comparison of the two samples via wavelength selection (basically, the emission wavelength depends on size *and* the degree of oxidation [24], which is different in the two samples).

Also plotted as insets in Fig. 5 are the distributions obtained by plotting the mean lifetimes obtained from the monochromated data, using Eqs. 5 or 9 to fit the data, as a function of the PL intensity at that wavelength. Since for these decays the beta parameter is reasonably close to 1, there is fairly little difference between the mean lifetimes calculated with the two versions of the SE model and the distributions obtained in this manner appear similar. While these decays do not represent the “true” distribution of lifetimes due to non-radiative contributions to *I*_*PL*_, they can nevertheless give an indication of the lifetime distribution. For the small particles, we observe a peak at ~ 47 μs, whereas for the large NCs the peak is more symmetrical and centered around 220 μs.

### Frequency-Resolved Spectroscopy

We started by validating the FRS data from two test standards: the first one was an RC circuit and the second was a sample of fluorescent Eu-chelate-doped microspheres (Fisher Scientific). The RC circuit has a mono-exponential decay in which the FRS data matched Eq. 9 quite closely and peaked at 12.7 kHz, in agreement with the measured decay time constant of 78.9 μs. The Eu-chelate PL spectrum peaked at 650 nm with a decay time in the order of hundreds of microseconds, presenting a standard for the Si NCs. The luminescence also decayed nearly mono-exponentially with a lifetime of 670 μs. The FRS data was centered at ~ 1570 Hz with a width virtually equal to the response function (Eq. 18), which is fairly close to the observed TRS result. The difference (636 vs. 670 μs) might be due to the slightly non-exponential behavior of the decay coupled to the excitation method, as discussed further below.

**Fig. 6:**
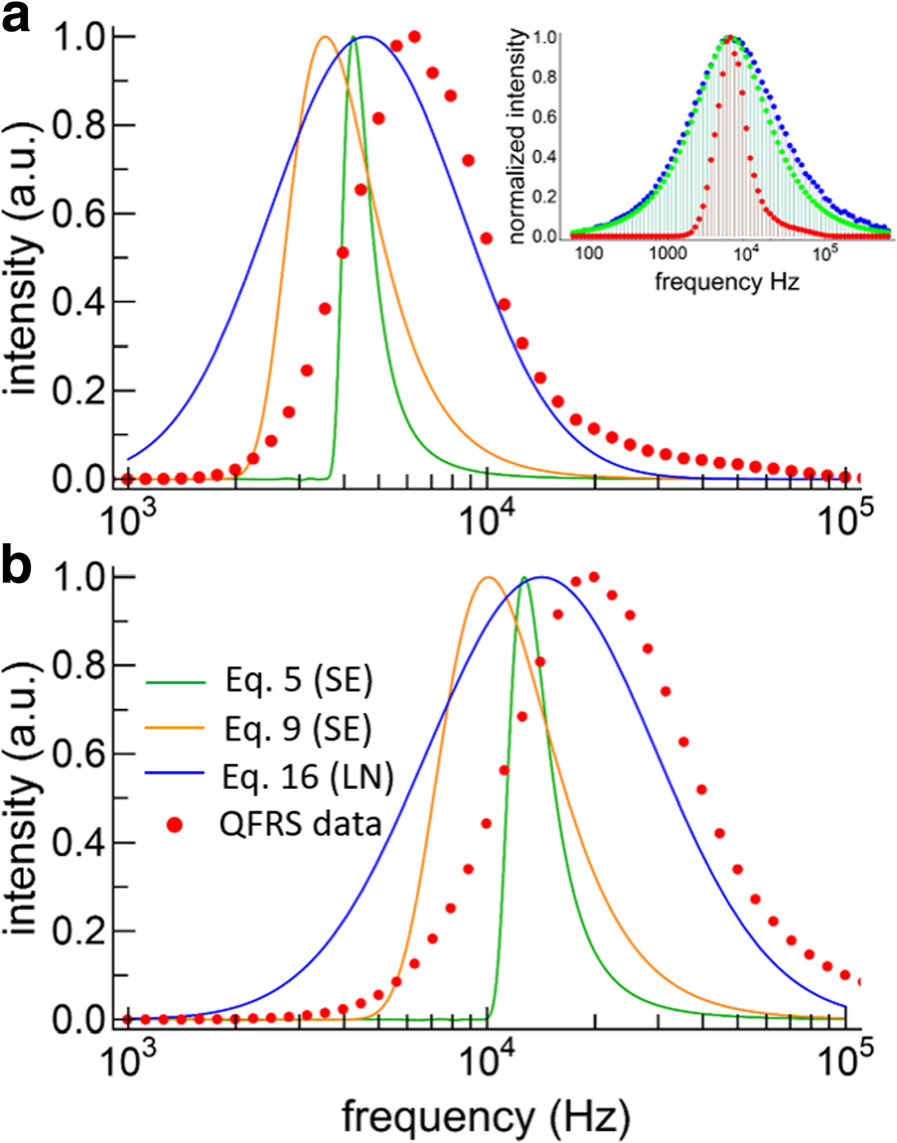
Lifetime distributions. **a** Lifetime distributions for large SiNCs obtained from fitting the TRS data with the two SE models and the LN model. The deconvolved QFRS data is also shown (red points). The inset shows the raw QFRS data for this sample (blue), the response function (green), and the deconvolution (red). **b** Lifetime distributions obtained by model fitting the TRS data (lines, same color scheme for both graphs) and QFRS (red points) for the small SiNCs

The FRS data for the Si-NCs is problematic because the observed QFRS results turned out to be only slightly broader than the response function (see the inset to Fig. 6a). Therefore, a deconvolution has to be performed on the data, which need to be nearly free of noise in order to avoid significant problems with the deconvolution procedure. We used the Richardson-Lucy deconvolution method [37] in order to enforce a positivity constraint. The deconvolved and normalized QFRS data then yield the measured lifetime distribution directly, as shown in Fig. 6 for the large and small NCs, respectively (red points), without assuming any model a priori. For both samples, we find a broad lifetime distribution that, in the case of the large NCs, is slightly skewed toward higher frequencies, whereas the small NCs distribution is more nearly symmetrical on a semilog plot. The decay rate distribution peaked at 19,900 Hz (50.3 μs) for the small NCs, whereas for the larger NCs the distribution peaked at 6280 Hz (159.2 μs).

The lifetime distributions obtained from the stretched exponentials (orange and green curves) and lognormal (blue curve) model fits are also plotted in Fig. 6 for the large and small particles. The three decay models yield different distributions, both in terms of the overall shape and the peak frequencies. For both samples, the QFRS peaks at a higher frequency than any of the TRS model fits. While this may seem surprising, the same effects have been observed for CdSe NCs having a distribution of lifetimes [38, 39]. In fact, the TRS decay curve for CdSe NCs was evidently sensitive to the pulse duration, with shorter pulses accentuating the shorter lifetimes and the opposite case for long pulses. Furthermore, the mean lifetimes obtained by long-pulse duration techniques were a factor of 3–4 times longer than those obtained by phase measurement, which was due to preferential excitation of the long-lived population in steady-state excitation [38]. Indeed, the response function for TRS with a slow repetition rate is narrower than for FRS, cutting off especially sharply on the high frequency side [29]. Essentially, FRS accentuates the short-lived components of the ensemble decay more than steady-state TRS does, and this may account for the difference in the peak frequencies obtained by TRS model fitting and FRS. Despite these inherent differences, FRS appears suited to uncovering the distribution of lifetimes in ensembles of SiNCs, because it is obtained by direct measurement rather than by an assumed model. For SiNCs typical of a thermally grown ensemble, the main drawback of FRS is the necessity of a deconvolution.

While the detector response function certainly affects the QFRS, it plays a role in the TRS data as well. Indeed, measuring the ensemble decay with the APD vs. the PMT setup yielded mean decay times that were different by a factor of ~ 2, regardless of the fitting model applied. The detector responsivity also affects choice of the TRS “best” model fit. As mentioned above, our Thorlabs APD responsivity peaks at 600 nm, whereas for our Hamamatsu PMT the responsivity maximizes at 850 nm, in the long-wavelength, slow-decay part of the SiNC spectrum. Although apparently not reported before in the literature on SiNCs, this issue means that wide-spectrum TRS results from different setups are not comparable. Unfortunately, despite some critical conclusions, ref. [38] also used different detectors to compare the decay dynamics from the same wide-band NC sample and the response functions may not have been the same. Fortunately, however, the phase measurements and the steady-state measurements used the same detector (as was the case here) and the differences in the observed dynamics for these situations remain valid. Finally, the detector response function is in principle correctable in the FRS data if the responsivity curve and monochromated decay rate distribution are known over a wide range of wavelengths (i.e., decay rates). The responsivity correction has no such simple solution with TRS alone.

## Conclusions

The most common models used for SiNC luminescence decay were described theoretically. The population decay corresponding to the “simple” stretched exponential luminescence decay, exp[− (*t*/*τ*)^*β*^], was derived and expressions for the characteristic mean times were found. This model was compared against the alternative model in which the population decays according to the simple SE. Two dodecene-functionalized SiNCs samples were then prepared from thermal nucleation and growth, followed by etching and alkane surface functionalization. These samples consisted of particles with mean diameters of 2.9 and 5.4 nm, respectively. The basic PL spectrum and TRS was measured using standard methods. The TRS data were fit with several distributions in order to establish whether any of them can be considered “true” and to find which one yields the best fit. While the simple SE luminescence decay fits the TRS data reasonably well, the distribution of residuals shows that it is not strictly accurate. None of the fitting models fully captures the shape of the measured decay rate distribution; they also show large deviations in the peak position and the shape of the distribution, as well as disagreement in the average time constants. Furthermore, the ensemble mean time constants were dependent on the responsivity curve of the detection system. This leads to serious questions about how to interpret the PL decay from ensembles of thermally-grown SiNCs.

Quadrature frequency-resolved spectroscopy was then employed with the intent to find the lifetime distribution directly for SiNC ensembles formed by thermal annealing of a base oxide. The spectrum was found to be not much wider than the intrinsic QFRS response function, requiring a deconvolution in order to extract the SiNC rate distribution. This yielded a distribution whose shape was nearly symmetrical (on a semilog scale) for the small NC sample and about half a decade wide, whereas it was slightly more skewed for the large NCs. We find that FRS techniques are suited to the study of SiNC luminescence dynamics and, after deconvolving the system response from the data, FRS yields the decay rate distribution directly. The most significant problem is the required deconvolution, but the Richardson-Lucy method was found to produce fairly robust results. While the detector response function can in principle be corrected from the FRS data, there is no simple means to do this for wide-PL-band TRS data. Still, as long as the data compared are from the same detector then the results should at least be internally meaningful. Hopefully in the future, these issues will be more fully considered when analyzing inhomogeneously broadened NC luminescence lifetimes, rather than defaulting to the simple stretched exponential model (Eq. 9) to describe and characterize the dynamical processes at work in the PL spectrum.

## Methods

The SiNCs were synthesized according to a recently-proposed method [21]. Briefly, 4 g of hydrogen silsesquioxane (HSQ) was annealed at 1100 or 1200 °C for 1 h in a flowing 5% H_2_ + 95% Ar atmosphere, resulting in composites of SiNCs embedded in a silica matrix. These composites were mechanically ground into a fine powder using an agate mortar. The powder was shaken for about 8 h with glass beads using a wrist action shaker. The powders were suspended in 95% ethanol and interfaced to a vacuum filtration system equipped with a filter. To liberate the H-SiNCs, the silica matrix was removed via HF etching. An approximately 200 mg aliquot of the composite was transferred to a Teflon beaker to which 2 mL of ethanol, 2 mL of water, and 2 mL of 49% HF aqueous solution were added in order to dissolve the silica matrix. After stirring the suspension for 40 min, the liberated H-SiNCs were extracted as a cloudy yellow suspension using toluene and isolated by centrifugation at 3000 rpm for 5 min. The resulting hydrogen-terminated SiNCs were suspended in 10 mL dry toluene, and then transferred to an oven-dried Schlenk flask equipped with a magnetic stir bar. Subsequently, 1 mL of 1-dodecene (ca. 4.6 mmol), as well as 20 mg of AIBN were added. The suspension was subjected to three freeze-pump-thaw cycles using an Ar charged Schlenk line. After warming the suspension to room temperature, it was stirred for 24 h at 70 °C, and 10 mL of methanol and 20 mL of ethanol were subsequently added to the transparent reaction mixture. The resulting cloudy suspension was transferred to a 50 mL PTFE vial and the SiNCs were isolated by centrifugation at 12,000 rpm for 20 min. The SiNCs were re-dispersed in 10 mL toluene and isolated by addition of 30 mL ethanol antisolvent followed by another centrifugation. The latter procedure was carried out one more time. Finally, the dodecyl-SiNCs were re-dispersed in 5 mL dry toluene and stored in a screw capped vial (concentration ~ 0.5 mg/mL) for optical studies.

TEM samples were prepared by depositing the freestanding nanoparticles directly onto an ultrathin (ca. 3 nm) carbon-coated copper TEM grid. The NCs were imaged by bright-field TEM using a JEOL JEM-2010 and HRTEM was done on a JEOL JEM-ARM200CF. Fourier transform infrared spectroscopy (FTIR) was performed in a Nicolet 8700 from Thermo Scientific. X-ray photo-electron spectroscopy was measured in a SPECS system equipped with a Phoibos 150 2D CCD hemispherical analyzer and a Focus 500 monochromator. The detector angle was set perpendicular to the surface and the X-ray source was the Mg Kα line.

**Fig. 7:**
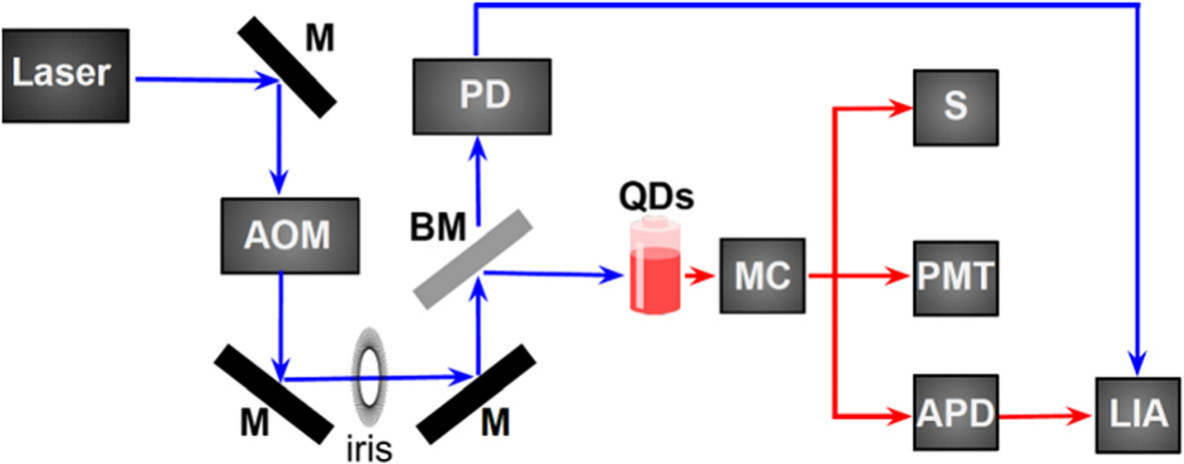
Diagram of the experimental setup. *M* mirror, *AOM* acousto-optic modulator, *BM* beamsplitter, *PD* photodiode, *MC* monochromator, *S* spectrometer, *PMT* photomultiplier tube, *APD* avalanche photodiode, *LIA* lock-in amplifier

Luminescence spectra were excited with a 352-nm Ar^+^ ion laser, which was pulsed (50% duty cycle, 50–250 Hz) using an Isomet IMDD-T110 L-1.5 acousto-optic modulator (AOM) with a fall time of ~ 50 ns. The used setup is schematically depicted in Fig. 7. The laser beam passes the acousto-optic modulator and one of the diffracted beams is selected by an iris. A beamsplitter reflects the main part of the pulsed laser beam into the sample cuvette and the incident power on the sample was ~ 8 mW spread over an area of ~ 4 mm^2^. The luminescence was collected with an optical fiber (numerical aperture 0.22), sent through a 450-nm longpass filter and is guided to the appropriate detector. The PL spectrum was measured by an Ocean Optics miniature spectrometer whose response function was corrected using a calibrated radiation source (the HL-3 + -CAL from Ocean Optics). The quantum efficiency was measured using an integrating sphere with 405-nm excitation, using a solution diluted to have an absorbance of ~ 0.15 at that wavelength.

The luminescence dynamics were measured with two different detectors. The first detector was the Thorlabs 120A2 avalanche photodiode (50 MHz roll-off), which was interfaced to a Moku:Lab (200 MHz) in digital oscilloscope mode. The second detector was a Hamamatsu h7422-50 photomultiplier tube interfaced to a Becker-Hickl PMS400 multiscalar. The error in the luminescence decay times was obtained by repeating the measurements three times, yielding a standard error in the mean lifetime calculated using the stretched exponential fit (Eq. 4) of 1 μs. All fits to the decay data were done in Origin using the least linear squares with the Levenberg-Marquardt algorithm, and were repeated in Matlab using the same method. For wavelength-dependent decay measurements, the luminescence was sent through an Acton MS2500i monochromator prior to detection, with the half width of the detected radiation set to ~ 3 nm.

For QFRS measurements, the AOM was set to produce a sinusoidal oscillation. A part of the incident beam was deflected into a Thorlabs PDA10A photodiode (200 MHz) in order to generate the reference signal. The SiNC PL response was simultaneously collected and sent to the APD. The reference signal was obtained using the beamsplitter, and along with the corresponding PL signal, was analyzed using the Moku:Lab in the lock-in amplifier mode to measure the in-phase and quadrature components of the signal.

Finally, we also searched for a short-lifetime component in the luminescence, as has sometimes been reported previously and attributed to oxidation [22]. This system used a 405-nm picosecond diode laser (Alphalas GmbH) to excite the NCs, and a Becker-Hickl HPM-100-50 PMT interfaced to an SPC-130 pulse counter system. This setup has a response time of ~ 100 ps. No evidence of a nanosecond decay was observed in these SiNCs.

## Additional File

Additional file 1: **Figure S1**. Normalized FTIR spectra of the synthesized SiNCs. The IR spectra and thus the surface composition is similar. However, it can be seen that the small SiNCs are more oxidized than the large particles (higher ratio of silicon oxide to CH_3_ bands). **Figure S2.** XPS spectra for the Si 2p orbital of the investigated particles. The ratio of oxide species to elemental silicon is considerably smaller for the large SiNCs. The XPS spectra were referenced to the Carbon C1s peak at 284.8 eV. **Figure S3.** PL intensity plotted against the excitation power for the large SiNCs. An 352 nm Ar+ ion laser beam was used to excite the sample. **Table S1.** Excitation power dependence of the SiNCs fitting parameters obtained from Eq. 4. The decay time τ and the stretching factor *β* remains almost the same within the used excitation powers. (DOCX 81 kb)

## Abbreviations

APD: Avalanche photodiode
AQY: Absolute quantum yield
FRS: Frequency-resolved spectroscopy
LN: Lognormal
NCs: Nanocrystals
PL: Photoluminescence
PMT: Photomultiplier tube
QFRS: Quadrature frequency-resolved spectroscopy
SE: Stretched exponential
SiNCs: Silicon nanocrystals
TRS: Time-resolved spectroscopy
