# Supplementary material for: Reappraising the Luminescence Lifetime Distributions in Silicon Nanocrystals
Source: Nanoscale Res Lett. 2018 Nov 28;13:383. doi: 10.1186/s11671-018-2785-x (PMC6261909; doi:10.1186/s11671-018-2785-x)
Supplement: Supplementary file 1 — Figure S1. Normalized FTIR spectra of the synthesized SiNCs. The IR spectra and thus the surface composition is similar. However, it can be seen that the small SiNCs are more oxidized than the large particles (higher ratio of silicon oxide to CH3 bands). Figure S2. XPS spectra for the Si 2p orbital of the investigated particles. The ratio of oxide species to elemental silicon is considerably smaller for the large SiNCs. The XPS spectra were referenced to the Carbon C1s peak at 284.8 eV. Figure S3. PL intensity plotted against the excitation power for the large SiNCs. An 352 nm Ar+ ion laser beam was used to excite the sample. Table S1. Excitation power dependence of the SiNCs fitting parameters obtained from Eq. 4. The decay time τ and the stretching factor β remains almost the same within the used excitation powers. (DOCX 81 kb) [file 11671_2018_2785_MOESM1_ESM.docx]

**Reappraising the Luminescence Lifetime Distributions in Silicon** **Nanocrystals**

**Supporting Information**

**Basic characterization – IR and XPS spectroscopy**

The IR spectra provide a first check of the functionalization process. Both spectra show the characteristic C-H stretching mode around 2800-3000 cm^-1^ as well as Si-H bonds at 2100 cm^-1^ and Si-O-Si around 1065 cm^-1^. The higher intensity of Si‑O-Si to CH_3_ bonds for the smaller particle size indicates higher surface oxidation compared to the larger SiNCs. Generally, it can be seen that the hydrosylilation reaction is not yielding a full surface coverage, as there is some remaining Si-H bonds visible.

Fourier transform infrared spectroscopy (FTIR) was performed in a Nicolet 8700 from Thermo Scientific.


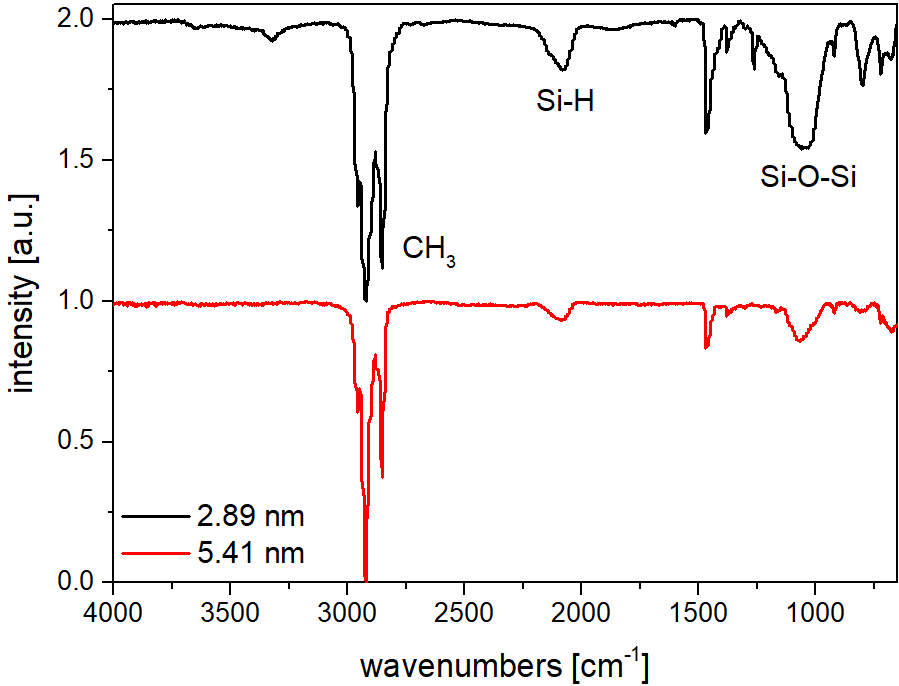


**Figure S1 Normalized FTIR spectra of the synthesized SiNCs.** The IR spectra and thus the surface composition is similar. However, it can be seen that the small SiNCs are more oxidized than the large particles (higher ratio of silicon oxide to CH_3_ bands).

To gain further insight about the degree of oxidation, XPS spectra were taken. In the following, high resolution XPS spectra for the different samples are shown. The small SiNCs show a higher ratio of oxide species compared to the large SiNCs further approving the result from FTIR. The oxide species are very likely produced during the synthesis process, as some steps cannot be done under inert atmosphere (e.g. HF etching with subsequent particle separation).

X-ray photo-electron spectroscopy was measured in a SPECS system equipped with a Phoibos 150 2D CCD hemispherical analyzer and a Focus 500 monochromator. The detector angle was set perpendicular to the surface and the X-ray source was Mg Kα.


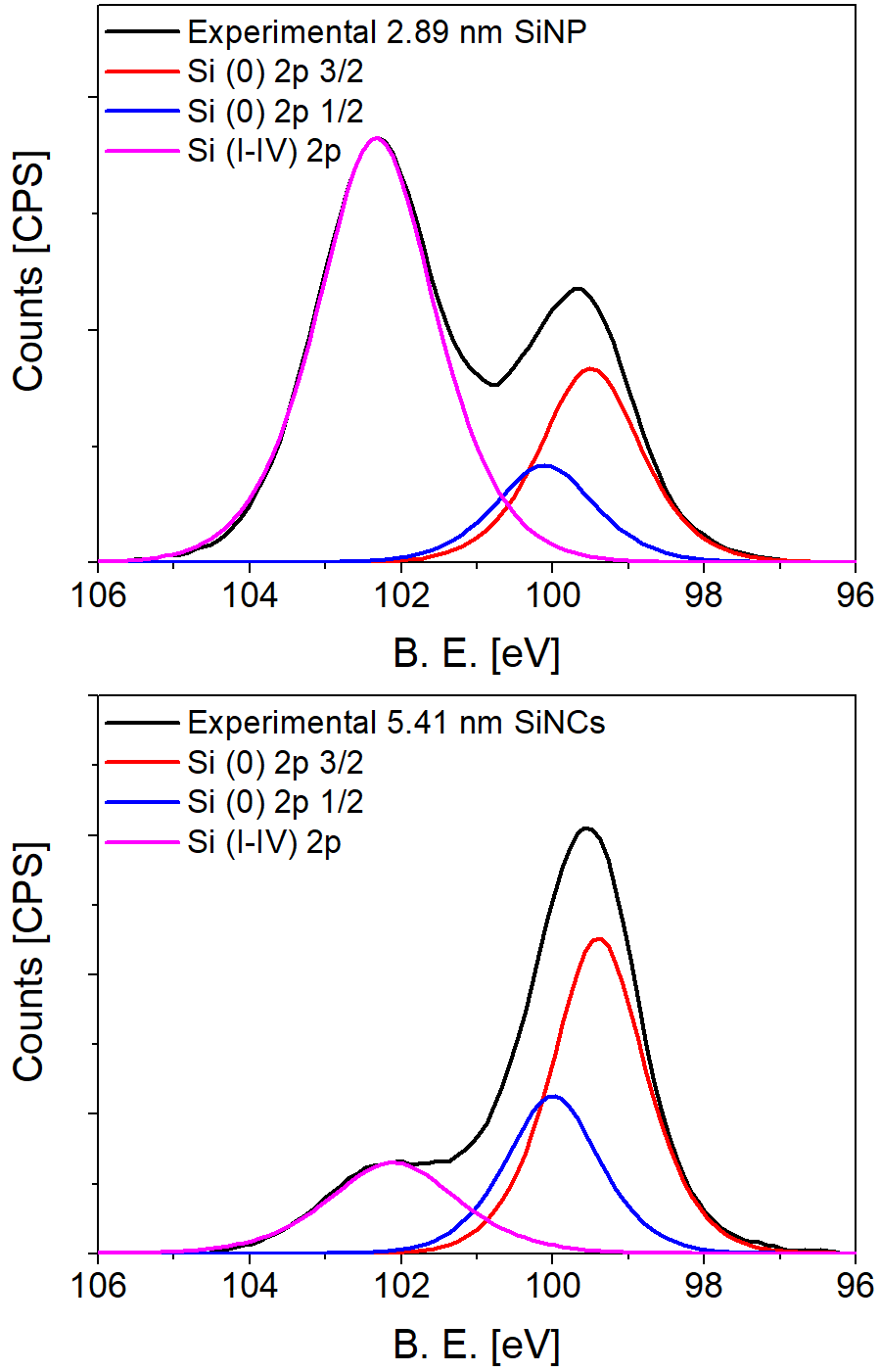


**Figure S2 XPS spectra for the Si 2p orbital of the investigated particles.** The ratio of oxide species to elemental silicon is considerably smaller for the large SiNCs. The XPS spectra were referenced to the Carbon C1s peak at 284.8 eV.

**Excitation power dependency of the PL intensity and measured lifetime**

As stated in the main article, the number of excitations per NC ~1 for the larger NCs suggests that one may be in the over-excitation regime in which there are additional non-radiative effects, due to the presence of multi-excitons on some NCs. In order to further evaluate this possibility, the lifetime was measured as a function of excitation power, resulting in Fig. S3 and Table S1. Though showing a saturation behavior in the PL intensities with increasing excitation power, the obtained values of τ and *β* remain unaffected within the experimental error of 2.1%.

Due to low intensities for small excitation power the Hamamatsu h7422-50 photomultiplier tube interfaced to a Becker-Hickl PMS400 multiscalar was used, which is the reason for the measured lifetimes appearing longer than in the main article.

The 352 nm Ar^+^ ion laser beam was pulsed (50% duty cycle, 100 Hz) using an Isomet IMDD-T110L-1.5 acousto-optic modulator (AOM) with a fall time of ~50 ns.


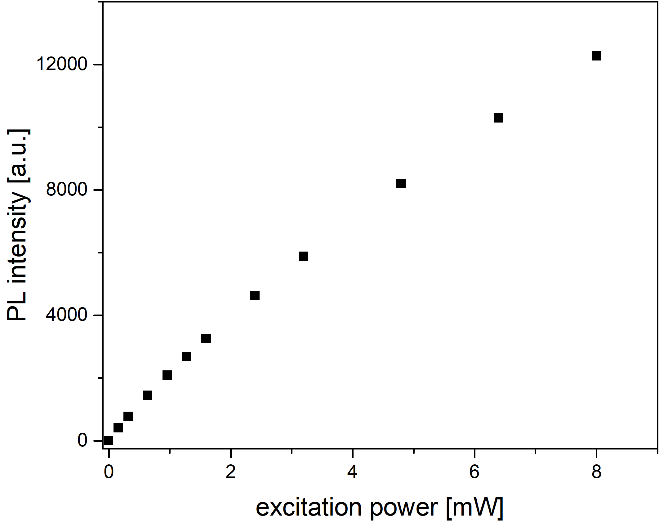


**Fig. S3:** PL intensity plotted against the excitation power for the large SiNCs. An 352 nm Ar+ ion laser beam was used to excite the sample.

**Table S1.** Excitation power dependence of the SiNCs fitting parameters obtained from Eq. 4. The decay time τ and the stretching factor *β* remains almost the same within the used excitation powers.

| excitation power | τ | *β* |
| --- | --- | --- |
| [mW] | [µs] | [a.u.] |
| 0.16 | 290.43 | 0.959 |
| 0.32 | 291.62 | 0.959 |
| 0.64 | 291.41 | 0.959 |
| 0.96 | 292.45 | 0.960 |
| 1.28 | 296.10 | 0.958 |
| 1.60 | 295.18 | 0.957 |
| 2.40 | 294.24 | 0.958 |
| 3.20 | 296.86 | 0.957 |
| 4.80 | 293.89 | 0.959 |
| 6.40 | 293.26 | 0.958 |
| 8.00 | 291.84 | 0.958 |
